# Lightweight Convolutional Neural Network and Its Application in Rolling Bearing Fault Diagnosis under Variable Working Conditions

**DOI:** 10.3390/s19224827

**Published:** 2019-11-06

**Authors:** Hengchang Liu, Dechen Yao, Jianwei Yang, Xi Li

**Affiliations:** 1 School of Mechanical-Electronic and Vehicle Engineering, Beijing University of Civil Engineering and Architecture, Beijing 100044, China; 2 Beijing Key Laboratory of Performance Guarantee on Urban Rail Transit Vehicles, Beijing University of Civil Engineering and Architecture, Beijing 100044, China; 3 Beijing Mass Transit Railway Operation Corporation Ltd, Beijing 100044, China

**Keywords:** rolling bearing, fault degree, lightweight network, fault diagnosis, working condition variation

## Abstract

The rolling bearing is an important part of the train’s running gear, and its operating state determines the safety during the running of the train. Therefore, it is important to monitor and diagnose the health status of rolling bearings. A convolutional neural network is widely used in the field of fault diagnosis because it does not require feature extraction. Considering that the size of the network model is large and the requirements for monitoring equipment are high. This study proposes a novel bearing fault diagnosis method based on lightweight network ShuffleNet V2 with batch normalization and L2 regularization. In the experiment, the one-dimensional time-domain signal is converted into a two-dimensional Time-Frequency Graph (TFG) using a short-time Fourier transform, though the principle of graphics to enhance the TFG dataset. The model mainly consists of two units, one for extracting features and one for spatial down-sampling. The building units are repeatedly stacked to construct the whole model. By comparing the proposed method with the origin ShuffleNet V2, machine learning model and state-of-the-art fault diagnosis model, the generalization of the proposed method for bearing fault diagnosis is verified.

## 1. Introduction

At present, with the rapid development of high-speed trains, the safety of trains under fast running conditions has become a serious problem. The train’s running gear is an important part of high-speed trains, and its operating status determines whether the train is safe. Rolling bearings are the most widely used part of the running gears, as well as the most vulnerable mechanical parts. Therefore, it is extremely important to monitor and diagnose the rolling bearing of the train’s running gear.

In recent years, a lot of research has been carried out on the condition monitoring and fault diagnosis of high-speed train running gears. Common monitoring methods include acoustic analysis [1], vibration analysis [2], temperature analysis [3], and so on. The vibration analysis method provides the best diagnosis and is the most widely used method. Vibration analysis usually consists of three steps: 1. acquire equipment vibration signals, 2. manually extract feature information from the signal, and 3. establish a model to diagnose faults by extracting the feature information [4].

With the era of big data, the amount of data has increased dramatically. Traditional diagnostic methods such as the maximum kurtosis spectral entropy deconvolution fault diagnosis proposed by Wang et al [5], and tri-axial vibration information fusion model proposed by Yang et al [6], highlight the problems of low diagnostic efficiency and the need for manual feature extraction. As a result, deep learning has been introduced into the field of fault diagnosis because it solves the problem of extracting features from nonlinear and nonstationary vibration signals, thereby avoiding complex feature engineering [7].

Most of the researches have realized the detection of bearing composite fault and fault degree through the analysis of vibration signals on experimental data or real-world data. The classical methods, such as Zhang et al. install accelerometers on the running parts of high-speed trains to collect vibration signals and use adaptive deep filtering technology to realize composite fault detection of train bearings [8]. Ding et al. verified that the proposed MQML-TQWT model can effectively detect early bearing failures on the wheelset-bearing system test bench [9]. Huang et al proposed MSVMD model which can automatically decompose the resonance frequency bands of the faulty bearing signal [10]. The DSLS-SVM proposed by Li et al. implements bearing fault diagnosis under various working conditions on public data sets and experimental data sets [11]. On the other side, deep learning methods such as Qiao et al. validated the effectiveness of AWMSCNN on the wheelset test bench and public datasets [12]. Liu et al present a comprehensive review of AI algorithms in rotating machinery fault diagnosis [13], Zhuang et al. proposed the SRDCNN model, which verified its workload adaptability and denoising ability under different working conditions [14].

Convolutional neural networks (CNNs) are the most common models in deep learning and have excellent performance in target detection and image classification tasks. Since the AlexNet won the ImageNet in 2012 [15], each session of the visual identity competition is a CNN model. CNN surpasses traditional machine learning in terms of development efficiency and classification accuracy. Due to the similarity between fault diagnosis and image classification, CNN has gradually been introduced into the field of fault diagnosis [16,17]. For example, Lu proposed to convert one-dimensional vibration signals into two-dimensional gray maps and then use CNN for classification [18]. Li proposed an intelligent fault diagnosis method for rolling element bearings based on the deep distance metric learning [19]. The above methods prove that deep learning has high precision and anti-noise in bearing fault diagnosis.

Although CNN performed well in the experiment, it has always faced problems in fault diagnosis: The model has poor generalization in different environments [20,21]. For example, in a real factory environment, the equipment usually runs at an inconsistent speed, and the bearing’s radial force and load torque are also different. In the case of fault monitoring, the model can only obtain good diagnostic results under conditions similar to the training data [22,23]. A lot of related research has been done on the generalization of the model, such as the bearing fault diagnosis based on the capsule network proposed by Zhu et al [24]. Zhang et al proposed a deep convolutional neural network with new training methods for bearing fault diagnosis [25]. For the traditional fault diagnosis method, the above research has improved the generalization of the model, but another problem has appeared—namely, the depth of the model is constantly increasing, resulting in the model size is too large.

Based on the analysis mentioned above, this paper proposes a bearing fault diagnosis method based on lightweight network ShuffleNet V2 [26], which replaces the classical convolutional layer with 1 × 1 convolution and depth-wise convolution and changes the convolution step to replace the pooled layer. The lightweight model still has strong generalization while reducing the size of the model and has higher diagnostic accuracy than previous studies. Common lightweight models include: MobileNet [27], SqueezeNet [28], Xception [29], and ShuffleNet [30]. ShuffleNet V2 is the most advanced lightweight model available and has better accuracy and speed than previous lightweight networks. The main contributions of this article include:

- Data augmentation is performed on the training set using the principle of graphics [31], which improves the size of the dataset and enhances the generalization ability of the model.
- Adding the Batch Normalization (BN) layer at the input and output positions of the model prevents the model from overfitting [32].
- The L2 regularization is added to the fully connected layer of the model to realize the function of weight attenuation, which reduces the overfitting to some extent.
- Comparing the proposed method with traditional machine learning and the other state-of-the-art CNN model, such as Vgg16 [33], ResNet [34], and ICN [24]. The experimental results show the excellent performance of the proposed method in model size and diagnostic accuracy.

The rest of the study is organized as follows: The theoretical background is given in Section 2. Section 3 details the structure of the proposed method. Section 4 compares the performance of some CNN models and traditional machine learning in two bearing fault datasets, proving the generalization of the method. Finally, we present our conclusions in Section 5.

## 2. Theoretical Background

In this section, we briefly introduce the structure of the lightweight model ShuffleNet V2 and the principle of batch normalization.

### 2.1. Batch Normalization

**Figure 1:**
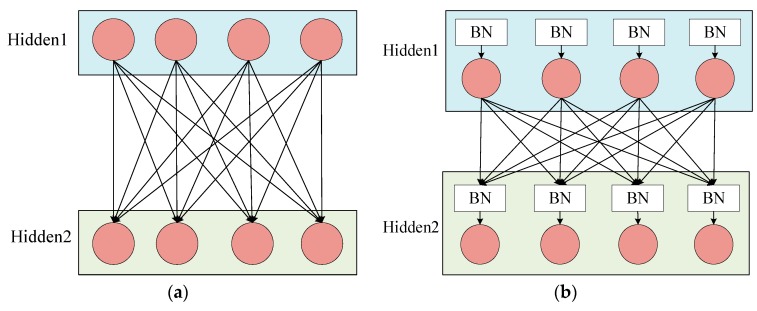
Hidden layer connection. (**a**) Normal connection. (**b**) Connection method using the Batch Normalization (BN).

In the training of the CNN model, since the input distribution of each layer changes continuously and the training time becomes longer, the setting of the learning rate and the initialization requirements of the model parameters are also strict, which makes it difficult to train the perfect nonlinear model. This problem is called the internal covariate shift. In order to solve this problem, researchers have proposed the BN method. The basic idea of batch normalization is to fix the input distribution of each hidden layer node. By using the theory of whitening in image processing, the input data distribution is transformed into a normal distribution with a mean of 0 and a variance of 1. Specifically, the BN layer is generally accessed after the activation value of the hidden layer is output. Figure 1a shows the hidden layer connection of the normal CNN model, and Figure 1b shows the hidden layer connection using BN.

Batch normalization can generally be simplified into two steps: 1. normalize each dimension, 2. scaling and shift of normalized data. Set a hidden layer input $x=(x^{\left(1\right)}\cdots x^{\left(d\right)})$, $E[x^{\left(k\right)}]$ represents the expectation of each neuron and $\sqrt{Var[x^{\left(k\right)}]}$ represents the standard deviation. The normalization formula is as follows:

(1) $${\hat{x}}^{\left(k\right)}=\frac{x^{\left(k\right)}-E[x^{\left(k\right)}]}{\sqrt{Var[x^{\left(k\right)}]}}$$

By normalizing the activation value of neurons, a normal distribution with a mean value of 0 and a variance of 1 is formed, which makes the input value of the nonlinear transformation function fall into the gradient unsaturated region, thus alleviating the problem of gradient disappearance, enhancing the information liquidity of backpropagation and the convergence speed of the network. However, it also leads to a non-linear reduction of the network, which leads to a decline in network expression capabilities. Therefore, it is necessary to perform scale and shift operations on each ${\hat{x}}^{\left(k\right)}$ after normalization to solve this problem. The expression is as follows:

(2) $$y^{\left(k\right)}=a^{\left(k\right)}{\hat{x}}^{\left(k\right)}+\beta^{\left(k\right)}$$

where $a^{\left(k\right)}$ is used for scale operation, $\beta^{\left(k\right)}$ is used for the shift operation.

When using mini-batch training, set the data in a mini-batch to $\varphi =\{x_{1\cdots m}\}$, and the values of α and β are continuously optimized during the training. The following shows the specific flow of Batch normalization. ε is a constant used to ensure the stability of the normalization operation.

Obtains the mean of the mini-batch:

(3) $$\mu_{\varphi }=\frac{1}{m}\sum_{i=1}^{m}x_{i}$$

Calculate the variance of mini-batch:

(4) $$\sigma_{\varphi }^{2}=\frac{1}{m}\sum_{i=1}^{m}{\left(x_{i}-\mu_{\varphi }\right)}^{2}$$

Normalize the input data:

(5) $${\hat{x}}_{i}=\frac{x_{i}-\mu_{\varphi }}{\sqrt{\sigma_{\varphi }^{2}+\varepsilon }}\;$$

Scale and shift:

(6) $$y_{i}=a{\hat{x}}_{i}+\beta$$

During training, the gradient of the loss ℓ and the gradient of the parameters associated with the BN transformation should be backpropagated using the following algorithm.

(7) $$\frac{\partial \ell }{\partial {\hat{x}}_{i}}=\frac{\partial \ell }{\partial y_{i}}\cdot \alpha$$

(8) $$\frac{\partial \ell }{\partial \sigma_{\varphi }^{2}}=\sum_{i=1}^{m}\frac{\partial \ell }{\partial {\hat{x}}_{i}}\cdot (x_{i}-\mu_{\varphi })\cdot \frac{-1}{2}{\left(\sigma_{\varphi }^{2}+\varepsilon \right)}^{-3/2}$$

(9) $$\frac{\partial \ell }{\partial \mu_{\varphi }}=(\sum_{i=1}^{m}\frac{\partial \ell }{\partial {\hat{x}}_{i}}\cdot \frac{-1}{\sqrt{\sigma_{\varphi }^{2}+\varepsilon }})+\frac{\partial \ell }{\partial \sigma_{\varphi }^{2}}\cdot \frac{\sum_{i=1}^{m}-2(x_{i}-\mu_{\varphi })}{m}$$

(10) $$\frac{\partial \ell }{\partial x_{i}}=\frac{\partial \ell }{\partial {\hat{x}}_{i}}\cdot \frac{1}{\sqrt{\sigma_{\varphi }^{2}+\varepsilon }}+\frac{\partial \ell }{\partial \sigma_{\varphi }^{2}}\cdot \frac{2(x_{i}-\mu_{\varphi })}{m}+\frac{\partial \ell }{\partial \mu_{\varphi }}\cdot \frac{1}{m}$$

(11) $$\frac{\partial \ell }{\partial \alpha }=\sum_{i=1}^{m}\frac{\partial \ell }{\partial y_{i}}\cdot {\hat{x}}_{i}$$

(12) $$\frac{\partial \ell }{\partial \beta }=\sum_{i=1}^{m}\frac{\partial \ell }{\partial y_{i}}$$

Therefore, adding a BN layer to the model can set a larger learning rate to improve the training speed and convergence speed of the model, while the requirements for initializing the model parameters are also reduced. The BN layer also has a regularization effect, which is beneficial to improve the generalization of the model and can replace the conventional regularization means such as dropout. Previous studies have also proved the effectiveness of BN. For example, Wang et al. significantly shortened the convergence time during training by adding the BN layer to the SAE model and improve the generalization of the model. The model was verified on the bearing and gear datasets [35]. Santurkar et al. deeply studied the influence of BN on the network from theoretical analysis and experimental verification. The experimental results show that BN makes the optimization environment significantly smoother. This smoothness makes the gradient behavior more predictive and stable, allowing faster Training [36].

### 2.2. Shufflenet V2

Traditional CNN models include convolutional layers, pooled layers, and fully connected layers. The existence of large convolution kernels and pooling layers makes the model computationally large. The model depth and size are increasing to improve the accuracy of the model. For some specific application scenarios such as mobile devices, because of their limited performance, the model requires high precision and small size.

**Figure 2:**
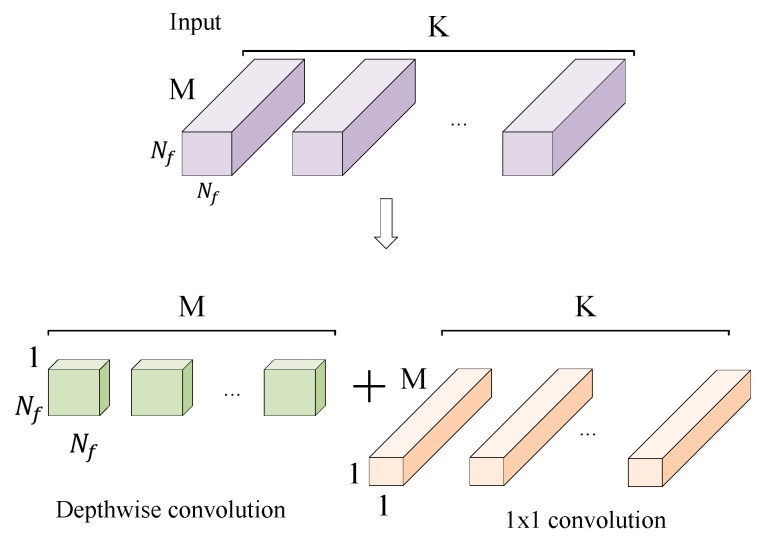
Depth-wise convolution and 1×1 convolution.

ShuffleNet V2 solves the above problems while avoiding the use of large convolution kernels and pooling layers. The traditional convolutional layer is replaced by a depth-wise convolution and a 1 × 1 small convolution kernel. As shown in Figure 2, the depth-wise convolution kernel size is 3 × 3, and one convolution kernel is responsible for one input channel, so the number of convolution kernels is the same as the number of input channels. 1 × 1 convolution is used to merge features of the output of depth-wise convolution. This improves the nonlinearity and enhances the expressive ability of the network without changing the size of the output feature graph. Instead of the traditional pooling layer, ShuffleNet V2 down-samples the feature by changing the depth-wise convolution step.

**Figure 3:**
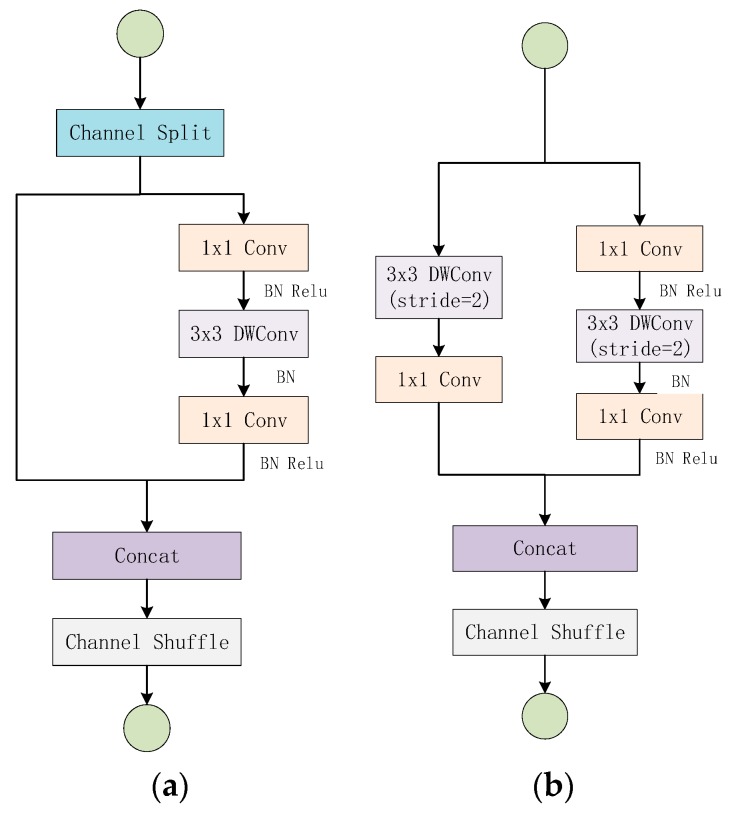
ShuffleNet V2 basic units: (**a**) basic unit, (**b**) basic unit for spatial down-sampling.

The ShuffleNet V2 network structure is mainly composed of two basic units stacked, as shown in Figure 3 [26]. The unit of Figure 3a uses “Channel Split”, “Channel Shuffle”, “Concat” to facilitate the exchange of feature information between different channels. At the beginning of the unit, it uses the “Channel Spilt” operation to divide the channel dimension of the input feature map into two branches equally, one branch remains unchanged, while the other branch contains three convolution layers. The output of the two branches is then combined by the “Concat” operation, and the number of channels output is the same as the number of channels of the input feature map. Finally, uses “Channel Shuffle” to disrupt the order of the output channels to ensure the exchange of feature information between the two branches. Unlike Figure 3a, Figure 3b removes the “Channel Split” operation, which doubles the number of output channels, and realizes the spatial down-sampling function by changing the depth-wise convolution step. ShuffleNet V2 is the same as other lightweight models by scaling the number of filters to change the complexity of the model, “ShuffleNet V2 s ×” means the complexity roughly $s^{2}$ times of ShuffleNet 1 ×, but in this paper we only consider ShuffleNet V2 1 × case. The overall structure of the network is shown in Table 1 [26]. Stage 2, Stage 3, and Stage 4 are all stacked by the cells in Figure 3. The Repeat column shows the number of stacks.

## 3. Details of the Proposed Method

Although the original ShuffleNet V2 model has excellent performance in image classification tasks, it cannot be directly applied to bearing fault diagnosis. Therefore, this section details our improvements in data preprocessing and network architecture to achieve a bearing fault diagnosis model with strong generalization.

### 3.1. Data Preprocessing

In the monitoring of equipment, the vibration data obtained are generally one-dimensional time-series signals, to utilize the ability of the CNN model to adaptively extract fault features, the one-dimensional vibration signal is usually converted into a 2-D format as an input to the model. For instance, Lu et al [18]. proposed used the 2-D gray-scale image which reconstructed by vibration signal image as the input of CNN model, but it has the problem that it can not reflect the frequency information of fault signal.

Fault signals generally have the characteristic of nonstationary and nonlinear. For the nonstationary signal, because its spectrum content changes greatly with time, the analysis method needs to accurately reflect the local time-varying spectrum characteristics of the signal. The traditional Fourier transform is the global transformation of the signal, which can’t analyze the change rule of the signal spectrum content with time.

Therefore, this paper proposes to convert 1-D time-series signals into 2-D TFG using Short-Time Fourier Transform (STFT). STFT is widely used in speech signal processing because it has superior performance in non-linear and non-stationary signal processing. The basic idea of STFT is to divide the non-stationary signal into several short-term stationary signals by a window function. After adding windows, the signal can be transformed using a Fourier transform, and the local spectrum in a small range near time t can be obtained. Therefore, compared with the traditional Fourier transform, STFT can obtain the range of specific frequency components in time domain. Equation (13) is STFT of signal z(t), and g(t) is the window function.

(13) $$STFT_{Z}(t,f)=\int_{-\infty }^{\infty }[z(t{}^{\prime })g(t{}^{\prime }-t)]e^{-j2\pi ft{}^{\prime }}dt{}^{\prime }$$

There are three main factors related to the STFT: the choice of the window function, the width of window and the number of points participating in STFT. The type of window function determines the magnitude of spectrum leakage and inter-spectral interference. The width of window affects the resolution of time domain and frequency domain. The number of points involved in the Fourier transform determines the resolution of time domain. The resolution in time domain and frequency domain can be derived from the following formula.

(14) $$T\;=\;\left\lfloor \frac{N_{x}-N_{o}}{N_{w}-N_{o}}\right\rfloor$$

(15) $$F=\left\{ \begin{array}{l} \frac{N_{f}}{2}+1,\;\;\;N_{f}\%2=0\\ \frac{N_{f}+1}{2},\;\;\;\;N_{f}\%2\neq 0\; \end{array}\right.$$

T stands for time-domain resolution and F stands for frequency domain resolution. $N_{x}$ denotes the sample length of the parameter STFT, $N_{f}$ denotes the number of points participating in the Fourier transform, $N_{w}$ denotes the window width, $N_{o}$ denotes the window overlap width. This paper uses Hanning window for short-time Fourier transform, setting $N_{x}$ to 1024, $N_{f}$ to 128, $N_{w}$ to 128, and $N_{o}$ to 114. Finally, the TFG with resolution of 65 × 65 is obtained.

**Figure 4:**
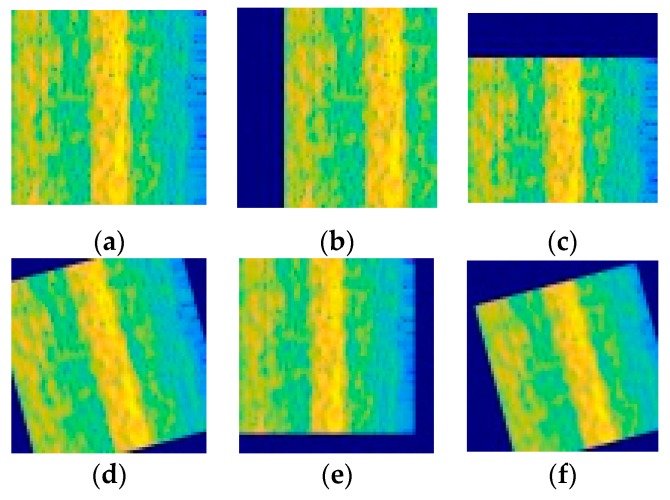
Data augmentation: (**a**) Original image, (**b**) Horizontal translation, (**c**) Vertical translation, (**d**) Rotation, (**e**) Zoom, (**f**) Zoom, translation and rotation.

Since the vibration data collected by the device under different working conditions have different characteristics, the time difference of the time spectrum obtained by the STFT is also large. If the model is trained directly using the TFG, the diagnostic accuracy of the model under different working conditions is poor. In order to solve the above problems, this paper proposes to use the horizontal translation, vertical translation, rotation, scaling and other operations in graphics to enhance the data, expand the dataset, and improve the generalization of the model, as shown in Figure 4.

The operation of image translation can effectively avoid the influence of data bias on the accuracy of the model. The blank part of the original image after translation is filled with a constant of 255 so that the image dimension after translation remains unchanged. Rotation operation refers to random rotation of the image in the range of 15 degrees. The zoom operation refers to random zooming in both the length and width directions. Shorten et al [37]. studied the effects of image data enhancement on deep learning in detail. The experimental results show that image data enhancement technology can avoid over-fitting of models and establish better data sets on popular open-source image datasets.

### 3.2. Improvement of Shufflenet V2

**Figure 5:**
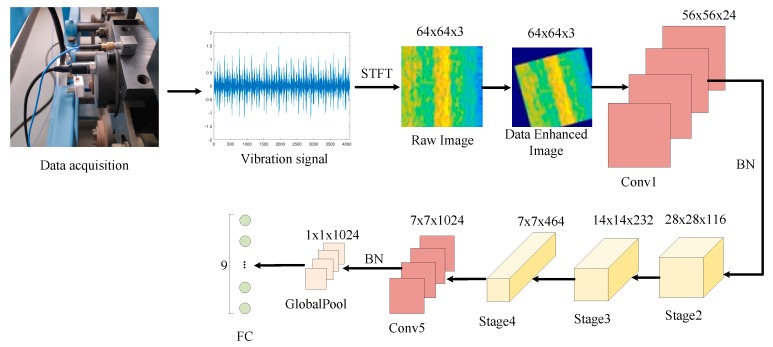
Flowchart of the proposed method.

As shown in Table 1, in the original Shufflenet V2 model, the dataset is ImageNet, and the input image size is 224 × 224. To reduce the size of the feature graph, a convolution layer and a maximum pooling layer are added to the initial position of the model. In this study, to make the characteristic graph and network structure consistent with the original Shufflenet V2 network, the convolutional layer and the pooling layer at the initial position are replaced by a convolutional layer (Conv1) with a convolution kernel of 9 × 9, and BN layer is added after the Conv1 and Conv5. The specific model structure is shown in Table 2, the time-frequency graph is input into the model in RGB format. Figure 5 shows how the network is worked.

For preventing the model from over-fitting on the test set. In the experiment, L2 regularization is added to the full connection layer. By adding the value of the original loss function and the square sum of the weight parameters, L2 regularization realizes the control of the weight preference of the model and finds the balance point between minimizing the original loss function and finding the small weight. Equation (16) is the cost function with L2 regularization, λ is the regularization parameter, n is the size of the training set, $C_{0}$ is the origin cost function.

(16) $$C=C_{0}+\frac{\lambda }{2n}\sum_{w}w^{2}$$

### 3.3. The Use of Optimizer

During the training period of the model, the optimizer continuously optimizes the value of loss function by updating and calculating the network parameters of the model, so that the model achieves the global optimal point. In practical application, the selection of loss function and optimizer determines the convergence speed and effect of the model. The inappropriate loss function and the optimizer will cause the model to fall into the local optimal point, which is the value of loss function hovers around the local optimal point, unable to reach the global optimal point, resulting in poor accuracy of the final model.

In this paper, the cross-entropy cost function is used to calculate the difference between the current model probability distribution and the real distribution to obtain the loss function value. Equation (17) is a formula for the cross-entropy cost function, where a is the output value of the neuron activation function and y is the desired output value.

(17) $$C=\;-\frac{1}{n}\sum_{x}\left[y\ln a+(1-y)\ln(1-a)\right]$$

For the purpose of avoiding the model falling into the local optimal point, this paper uses the RMSProp optimizer to optimize the model. RMSProp not only accelerates the convergence speed of the model but also avoids the problem of excessive swing of the loss function in the optimization process. When RMSProp optimizer is initialized, it is necessary to set the global learning rate ε, the decay rate ρ and constant δ. Suppose that one mini-batch in the training process contains m samples $\left\{x^{\left(1\right)},\cdots x^{\left(m\right)}\right\}$, corresponding labels to $y^{\left(i\right)}$. First, the gradient g needs to be calculated to get the cumulative square gradient r, then the parameter update Δθ is calculated, and finally the initial parameter θ is updated. In the experiment, ε was set to 0.001, ρ was set to 0.9, and the constant δ was set to prevent the numerical mutation from being set to $10^{-6}$ during the dividing operation. Algorithm 1 is the detailed flow of RMSProp optimizer.

**Algorithm 1** RMSProp Algorithm1: Initialize accumulation variables r=0
2: While stopping criterion not met do3: Sample a minibatch of m examples from the   training set $\left\{x^{\left(1\right)},\cdots ,x^{\left(m\right)}\right\}$ with corresponding   targets $y^{\left(i\right)}$
4: Computing gradient: $g\leftarrow \frac{1}{m}\nabla_{\theta }\sum_{i}L(f(x^{\left(i\right)};\theta ),y^{\left(i\right)})$
5: Accumulate squared gradient: r←ρr+(1−ρ)g⊙g 6: Compute parameter update: $\Delta \theta =-\frac{\varepsilon }{\sqrt{\delta +r}}⊙g$
7: Apply update: θ←θ+Δθ
8: end while

## 4. Experimental Verification and Analysis

In this section, the generalization of the proposed method will be verified by two published bearing fault datasets. The experiment hardware included an R5-2600X CPU, 16G RAM, and an RTX2080ti GPU. The dataset which consists of TFGs is made by the MATLAB software, the neural structure is developed by Keras, and the programming language is Python3.6. 

### 4.1. Case 1: Generalization on Different Loads in the Case Western Reserve University Dataset

In this test, only the data under a single load is used as the model training set, and the data under other loads is used as a test set to verify the generalization of the proposed method.

#### 4.1.1. Data Description

**Figure 6:**
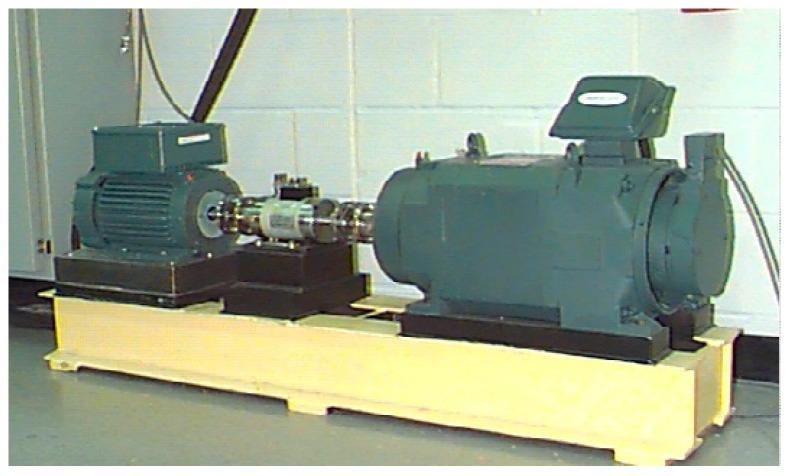
The test rig of Case Western Reserve University (CWRU).

The dataset consists of bearing data published by Case Western Reserve University (CWRU) [38]. The experimental platform consists of four units from left to right: a 2HP motor, a torque sensor, a dynamometer, and control electronic equipment. The accelerometer is mounted on a housing with a magnetic base and uses a 16 channel DAT recorder to collect vibration signals. During the experiment we use the vibration data collected by the drive-end acceleration sensor at a sampling frequency of 12 kHz. The data includes nine types of faults. The inner ring, outer ring and rolling elements of the bearing are collected at fault diameters of 7 mils, 14 mils, and 21 mils. Figure 6 is a picture of the test rig.

The training set, validation set, and test set are established for bearing fault data under each load. The training set includes 13500 TFGs, the validation set includes 450 TFGs and the test set includes 900 TFGs. The specific dataset composition is shown in Table 3.

#### 4.1.2. Introduction to Contrast Experiments

To show the superiority of the proposed method in accuracy and model size more clearly, we compared some models with good performance in fault diagnosis and image classification, including the famous k-Nearest Neighbor (kNN) and Support Vector Machine (SVM) in the field of traditional machine learning. In the experiment, the signals transformed by Fourier transform are used as input data of kNN and SVM. The contrast experiment also includes some deep learning models widely used in image classification, such as Vgg16, ResNet and the classic lightweight model MobileNet. Other comparative experiments include the latest diagnostic model neural network based on a capsule network with an inception block (ICN). Vgg16, MobileNet, ICN and the proposed method use the same TFG as the model input. During the experiment, the training time, the accuracy and size of the model are selected as the evaluation index.

#### 4.1.3. Diagnostic Results and Analysis

This section will highlight the strong generalization of the proposed model under different loads using tables and figures. In the process of model training, each epoch is predicted on the validation set, the model parameters, diagnostic accuracy, and loss values are saved. After training, we will use the model parameters which have the lowest loss value to predict the test set. 

**Figure 7:**
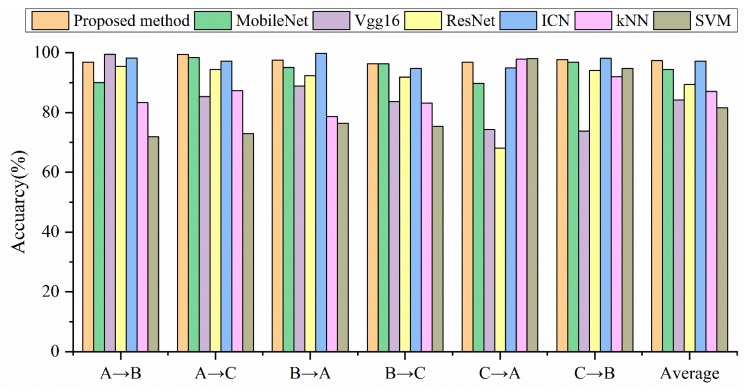
Accuracy comparison of different models under different loads.

Table 4 compares the training time of the proposed method and other deep learning models. The comparative experimental results are shown in Table 5 and Figure 7, where “A→B” means using Setting A as the model training set and Setting B as the model test set. 

**Figure 8:**
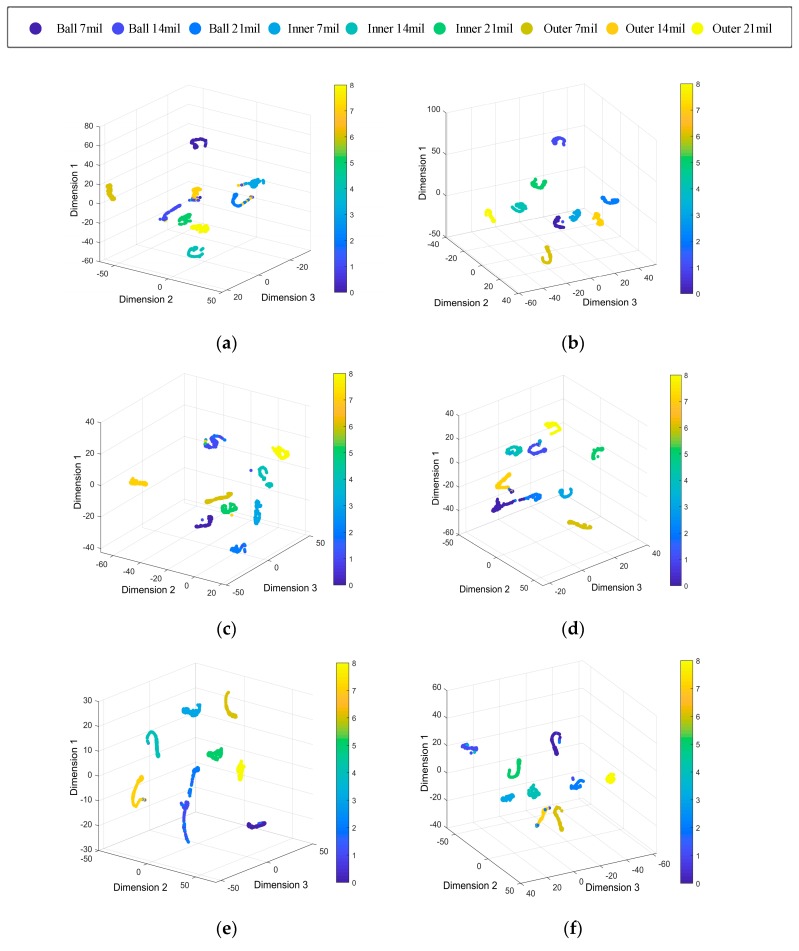
Feature visualization maps by the proposed method. (**a**) A→B, (**b**) A→C, (**c**) B→A, (**d**) B→C, (**e**) C→A, (**f**) C→B.

To better show the performance of the proposed method on different training sets and test sets, Figure 8 uses the t-SNE algorithm to visualize the prediction results of the model [39]. The T-SNE algorithm reduces the dimension of high-dimensional data, which is convenient to show the classification results in three-dimensional space and observe the effect of model classification. In the three-dimensional space after dimension reduction, the level indicators are dimensionless, and the distance in the spatial coordinates only represents the distance degree of different categories. Figure 8 shows that in the case of A→C, the diagnosis effect of the proposed method is the best, in other cases, most features clustering successful, which also proves that the proposed method can adaptively extract effective features to achieve an accurate diagnosis.

The experimental results show that the deep learning model is generally stronger than traditional machine learning in terms of model size and diagnostic accuracy. Table 4 shows that the proposed method has faster training time than other deep learning models. On the other hand, in terms of the average diagnostic accuracy, the proposed method is higher than all the comparison experiments. Although the diagnostic accuracy of ICN is close to the proposed method, the model size is 3.6 times that of the proposed method. From the perspective of model size, both the proposed method and MobileNet belong to the lightweight model, and the size of other models is at least three times that of the lightweight model. Although the model size of MobileNet is 3.5 MB smaller than the proposed method, the diagnostic accuracy is lower than the proposed method under any working conditions, and the average accuracy is 3.04% lower than the proposed method. In the case of A→C, the proposed method has the best performance, and the error rate is only 0.6%, while the ICN error rate is 2.83%, MobileNet error rate is 1.6%, and Vgg16 error rate is 14.7%. From the above analysis, we can see that the proposed method has good generalization on the CWRU dataset.

### 4.2. Case 2: Generalization on Different Loads in the Paderborn University Dataset

In this section, the generalization of the proposed method under different loads will be verified by the bearing fault dataset provided by Paderborn university. The model is trained with data under a single load and tested with data under other loads.

#### 4.2.1. Data Description

**Figure 9:**
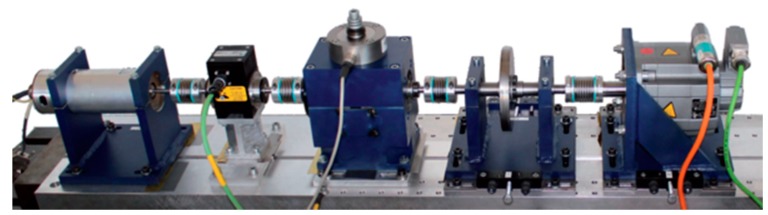
Test rig of Paderborn University.

The dataset is provided by the Chair of Design and Drive Technology, Paderborn University, Germany. The dataset includes vibration and motor current signals for condition monitoring [40]. As shown in Figure 9, the test rig consists of a test motor, measuring shaft, bearing module, flywheel, and load motor. The experimental bearing type is 6203. The vibration sensor is model no. 336c04 and the measuring hardware is processor board DS 1006 single-core produced by dSPACE GmbH, the force measurement equipment is measuring box K11 from Laurents messthnik.

In this paper, the vibration signal at 1500 rpm is used. Load torque is 0.7 Nm or 0.1 Nm, the radial force is 1000 N or 400 N, the sampling frequency is 64 kHz. Depending on the operating conditions, the dataset consists of three parts: Setting D, Setting E, Setting F. as shown in Table 6. Each part of the training set contains 9000 TFGs, the validation set includes 450 TFGs, and the test set includes 900 TFGs.

#### 4.2.2. Diagnostic Results and Analysis

**Figure 10:**
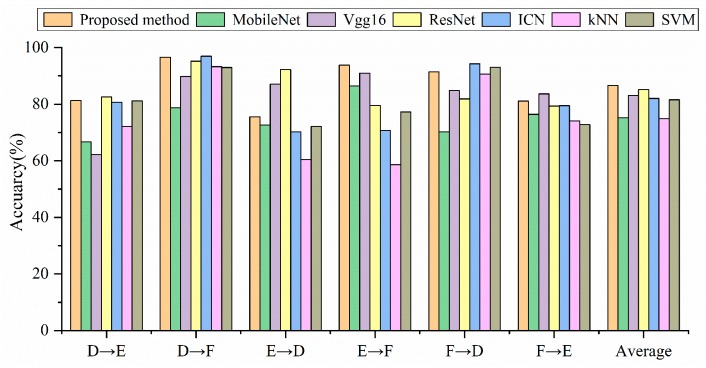
Accuracy comparison of different models under different loads.

In this section, the generalization of the proposed method will be verified in Table 7 and Figure 10. Unexpectedly, the diagnostic accuracy of the SVM in the case of F→D exceeds the proposed method, and it becomes the most accurate model on the Paderborn dataset. We think this might be because the signal transformed by the Fourier transform is directly used as the input of SVM. The traditional machine learning algorithm needs to re-select and extract features in different environments. The selection of features determines the accuracy of the machine learning model.

The average diagnostic accuracy of the proposed method is still the highest on the Paderborn dataset. The average accuracy of MobileNet is 11.43% lower than that of the proposed method. The average accuracy of Vgg16 and ICN is 3.52% and 4.57% lower than the proposed method. The proposed method performs best in the case of D→F, with an accuracy of 96.55%.

Table 8 shows the training time for the proposed method and other deep learning models on the Paderborn university dataset. The training time of the proposed method is faster than any other deep learning model.

**Figure 11:**
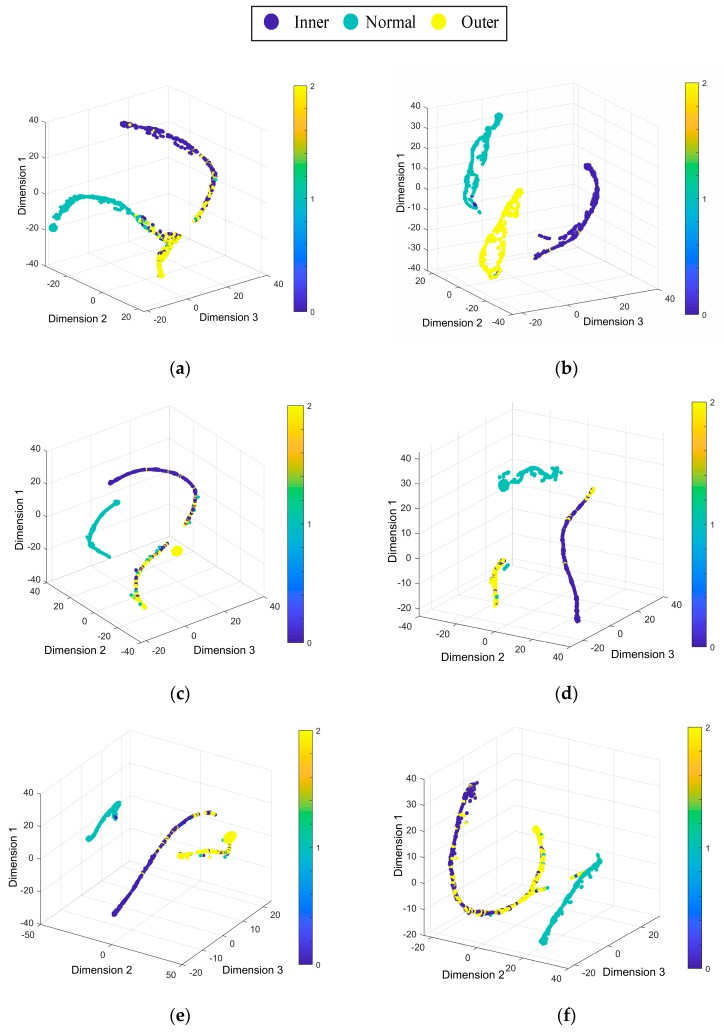
Feature visualization maps by the proposed method: (**a**) D→E, (**b**) D→F, (**c**) E→D, (**d**) E→F, (**e**) F→D, (**f**) F→E.

Figure 11 uses the t-SNE algorithm to reduce the data dimension of the prediction results [39], showing the diagnosis effect of the proposed method on the Paderborn University dataset. Figure 11 shows that the model has the best diagnosis effect in the case of D→F, and the diagnosis effect is unsatisfactory in the case of E→D. the health states outer is mixed with the other health states. It is obvious that the deep learning model has a lower diagnostic effect on the Paderborn dataset than the CWRU dataset. This might be because the data quality of CWRU dataset is better than Paderborn dataset.

### 4.3. Analysis of Cost Function optimization

**Figure 12:**
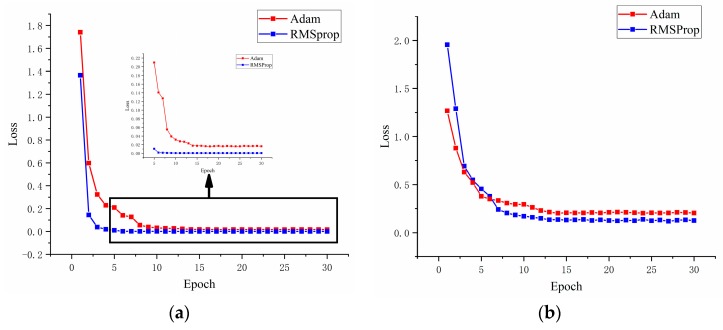
RMSProp optimizer and Adam optimizer optimize the cost function on different datasets: (**a**) C→B (CWRU dataset), (**b**) D→E (Paderborn university dataset).

At present, most CNN models use Adam optimizer to optimize the cost function, and Adam optimizer generally shows excellent performance [41,42]. However, in the lightweight model, the performance of Adam optimizer is unsatisfactory, so the proposed method uses the RMSProp optimizer to optimize the model. Figure 12 shows the optimization process of RMSProp optimizer and Adam optimizer on different datasets. Figure 12a is the cost function optimization process during the C→B experiment on the CWRU dataset, and Figure 12b is the optimization process during the D→E experiment on the Paderborn University dataset.

Figure 12 shows that the optimization effect of RMSProp optimizer on cost function is significantly better than that of Adam optimizer. When the initial loss value is large, the optimization speed and effect of RMSProp optimizer are also satisfactory.

### 4.4. Analysis of the Proposed Method

**Figure 13:**
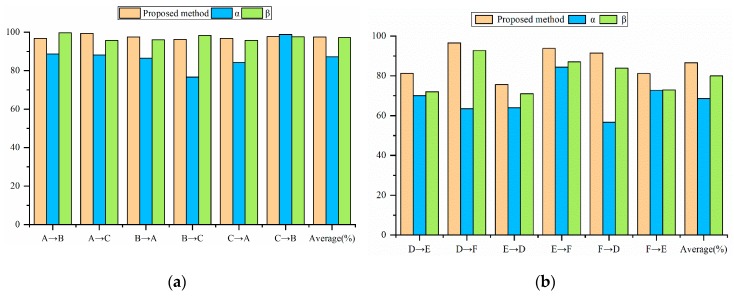
Comparison of different models on the bearing fault dataset: (**a**) Comparison of different models on CWRU dataset, (**b**) Comparison of different models on the Paderborn dataset.

In order to verify the effect of data augmentation, BN layer and L2 regularization in the proposed method, this section will compare the proposed method with the original ShuffleNet V2 using different improved methods. Model α is the original ShuffleNet V2 model. Model β is the original ShuffleNet V2 using data augmentation. Table 9 and Table 10 show the diagnostic effects of different models on the CWRU dataset and the Paderborn dataset. Figure 13a shows different models using different load data for testing on the CWRU dataset. Figure 13b shows different models using different case data for testing on the Paderborn dataset.

From the perspective of average diagnostic accuracy, the proposed method outperforms other comparison models on both datasets. The average diagnostic accuracy of the proposed method on the CWRU dataset is 10.28% which is 0.31% higher than that of the Model α and the Model β, respectively. The average accuracy of the proposed method on the Paderborn dataset is 17.35% and 6.43% higher than the Model α and the Model β. It is worth noting that the proposed method has lower accuracy than the Model β in the case of A→B and B→C on the CWRU dataset. This might be because of the similarity of data in CWRU datasets under different loads, and in some cases using data augmentation will reduce the accuracy of the model.

**Figure 14:**
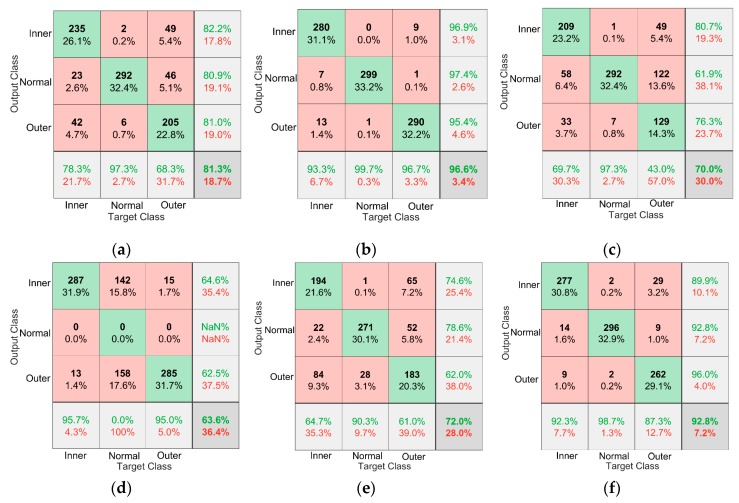
Confusion matrix of different models in the case of A→B and A→C. (**a**) D→E Proposed method, (**b**) D→F Proposed method, (**c**) D→E Model α, (**d**) D→F Model α, (**e**) D→E Model β, (**f**) D→F Model β.

To evaluate the effectiveness of the models in diagnosing different types of faults, a confusion matrix made by MATLAB is used to visualize the diagnostic results of different models. The green block in the image represents the number of correctly predicted samples, the red block represents the number of incorrectly predicted samples, the percentage in the color block shows the percentage of the current block in the total number of samples, and the block in the lower right corner of the matrix shows the accuracy of the model. Each row of the matrix represents the instances in an output class while each column represents the instances in an actual class. The rightmost column represents the target of the output class, and the last row represents the recall rate. Figure 14 shows a representation of the proposed method, Model α, and Model β in the case of D→E and D→F on the Paderborn dataset.

It can be seen from Figure 14d,f that the use of data augmentation solves the problem that Model α cannot correctly diagnose normal bearings, and the diagnostic accuracy of normal bearings is improved from 0% to 96.7%. Figure 14b,f show that the use of the BN layer and L2 regularization significantly improves the diagnostic accuracy of the model for bearing operating conditions. Figure 14 shows that the proposed method is superior to other comparison models in the diagnostic accuracy of bearings in different states.

## 5. Conclusions

By improving the existing ShuffleNet V2 model, a bearing fault diagnosis model with good generalization is obtained, and the proposed method is verified on two public datasets. Through the experiments described in this paper, we can derive the following conclusions:

1) The method proposed in this study not only improves the accuracy of the model but also greatly reduces the size of the model, illustrating the lightweight design of the model.
2) The traditional machine learning model can still achieve similar performance with deep learning when extracting appropriate features. But in different environments, feature selection needs to be repeated since otherwise, the diagnostic accuracy will decrease significantly.
3) Through data augmentation of the network input image, adding BN layer and L2 regularization in the network, the diagnostic accuracy of ShuffleNet V2 for bearings under different conditions can be effectively improved, and the model has strong generalization ability.

In future work, we will continue to explore how to reduce the size of the model while improving its accuracy, so that deep learning can be better applied to the field of bearing fault diagnosis.

## Figures and Tables

**Table 1:** Overall structure of shuffleNet V2.

Layer	Output Size	Kernel Size	Stride	Repeat	Output Channels
Image	224 × 224				3
Conv1 MaxPool	112 × 112 56 × 56	3 × 3 3 × 3	2 2	1	24
Stage2	28 × 28 28 × 28		2 1	1 3	116
Stage3	14 × 14 14 × 14		2 1	1 7	232
Stage4	7 × 7 7 × 7		2 1	1 3	464
Conv5	7 × 7	1 × 1	1	1	1024
GlobalPool	1 × 1	7 × 7			
FC					1000

**Table 2:** ShuffleNet V2 for Bearing Fault Diagnosis.

Layer	Output Size	Kernel Size	Stride	Repeat	Output Channels
Image	64 × 64				3
Conv1	56 × 56	9 × 9	1		24
BN	56 × 56				
Stage2	28 × 28 28 × 28		2 1	1 3	116
Stage3	14 × 14 14 × 14		2 1	1 7	232
Stage4	7 × 7 7 × 7		2 1	1 3	464
Conv5	7 × 7	1 × 1	1	1	1024
BN	7 × 7				
GlobalPool	1 × 1	7 × 7			
FC					9

**Table 3:** Dataset composition of CWRU.

Motor Speed (rpm)	Motor Load (HP)	Fault Diameter (mils)	Name of Setting
1772	1	7,14,21	A
1750	2	7,14,21	B
1730	3	7,14,21	C

**Table 4:** Training time for the proposed method and other deep learning models.

Model	Training Time (s)
Proposed method	805.6
MobileNet	978.4
Vgg16	810.8
ResNet	1401.1
ICN	1856.0

**Table 5:** Different model accuracy and size comparison on the CWRU dataset.

Model	A→B	A→C	B→A	B→C	C→A	C→B	Model Size (MB)	Average (%)
Proposed method	96.80	99.40	97.55	96.30	96.30	97.80	16	97.42
MobileNet	90.00	98.40	95.00	96.30	89.80	96.80	12.5	94.38
Vgg16	99.50	85.30	88.80	93.60	74.30	73.70	58.5	84.20
ResNet	95.44	94.33	92.33	91.88	68	93.99	20.3	89.33
ICN	98.23	97.17	99.80	94.71	94.93	98.10	56.9	97.15
kNN	83.27	87.33	78.57	83.17	97.80	91.97	875	87.02
SVM	71.93	72.90	76.33	75.30	98.03	94.77	145	81.55

**Table 6:** Different model accuracy and size comparison on the CWRU dataset.

Rotating Speed (rpm)	Running State	Load Torque (Nm)	Radialforce (N)	Name of Setting
1500	health, inner fault, outer fault	0.1	1000	D
1500	health, inner fault, outer fault	0.7	400	E
1500	health, inner fault, outer fault	0.7	1000	F

**Table 7:** Different model test on the dataset from Paderborn University.

Model	D→E	D→F	E→D	E→F	F→D	F→E	Model Size (MB)	Average (%)
Proposed method	81.30	96.55	75.55	93.77	91.44	81.11	16	86.62
MobileNet	66.70	78.77	72.66	86.44	70.22	76.40	12.5	75.19
Vgg16	62.20	89.77	87.11	91.00	84.88	83.66	58.5	83.10
ResNet	82.55	95.22	92.22	79.55	81.88	79.33	20.3	85.13
ICN	80.67	96.97	70.23	70.67	94.27	79.50	53.3	82.05
kNN	72.13	93.27	60.47	58.60	90.67	74.13	594	74.88
SVM	81.20	92.97	72.13	77.23	93.00	72.77	281	81.55

**Table 8:** Training time of deep learning model on Paderborn University dataset.

Model	Training Time (s)
Proposed method	557.1
MobileNet	678.9
Vgg16	681.9
ResNet	910.0
ICN	569.6

**Table 9:** Different model test on the dataset from CWRU.

Model	A→B	A→C	B→A	B→C	C→A	C→B	Average (%)
Proposed method	96.80	99.40	97.55	96.30	96.30	97.80	97.42
α	88.70	88.11	86.5	76.66	84.11	98.77	87.14
β	99.70	95.70	96.00	98.30	95.70	97.66	97.16

**Table 10:** Different model test on the dataset from Paderborn University.

Model	D→E	D→F	E→D	E→F	F→D	F→E	Average (%)
Proposed method	81.30	96.55	75.55	93.77	91.44	81.11	86.62
α	70.00	63.55	64.00	84.33	56.70	72.60	68.53
β	72.00	92.77	71.00	87.00	83.77	72.88	79.90
